# Simultaneous Sensitive Determination of *δ*^13^C, *δ*^18^O, and *δ*^17^O in Human Breath CO_2_ Based on ICL Direct Absorption Spectroscopy

**DOI:** 10.3390/s22041527

**Published:** 2022-02-16

**Authors:** Ligang Shao, Jiaoxu Mei, Jiajin Chen, Tu Tan, Guishi Wang, Kun Liu, Xiaoming Gao

**Affiliations:** 1 Anhui Institute of Optics and Fine Mechanics, Hefei Institutes of Physical Science, Chinese Academy of Sciences, Hefei 230031, China; shao_ligang@126.com (L.S.); jjchen@aiofm.ac.cn (J.C.); tantu@aiofm.ac.cn (T.T.); gswang@aiofm.ac.cn (G.W.); liukun@aiofm.ac.cn (K.L.); 2 Science Island Branch of Graduate School, University of Science and Technology of China, Hefei 230026, China

**Keywords:** laser absorption spectroscopy, CO_2_ isotopes, breath analysis

## Abstract

Previous research revealed that isotopes ^13^C and ^18^O of exhaled CO_2_ have the potential link with *Helicobacter pylori*; however, the ^17^O isotope has received very little attention. We developed a sensitive spectroscopic sensor for simultaneous *δ*^13^C, *δ*^18^O, and *δ*^17^O analysis of human breath CO_2_ based on mid-infrared laser direct absorption spectroscopy with an interband cascade laser (ICL) at 4.33 μm. There was a gas cell with a small volume of less than 5 mL, and the pressure in the gas cell was precisely controlled with a standard deviation of 0.0035 Torr. Moreover, real-time breath sampling and batch operation were achieved in gas inlets. The theoretical drifts for *δ*^13^C, *δ*^18^O, and *δ*^17^O measurement caused by temperature were minimized to 0.017‰, 0.024‰, and 0.021‰, respectively, thanks to the precise temperature control with a standard deviation of 0.0013 °C. After absolute temperature correction, the error between the system responded *δ*-value and the reference is less than 0.3‰. According to Allan variance analysis, the system precisions for *δ*^13^C, *δ*^18^O, and *δ*^17^O were 0.12‰, 0.18‰, and 0.47‰, respectively, at 1 s integration time, which were close to the real-time measurement errors of six repeated exhalations.

## 1. Introduction

Breath analysis, which is a non-invasive and painless method, has been proven to have potential for disease screening and diagnosis [1,2,3,4,5]. Carbon dioxide (CO_2_) is one of the most important components in human exhaled substance, accounting for about 5%. The most abundant isotopologues of CO_2_ are ^12^C^16^O_2_, ^13^C^16^O_2_, and ^18^O ^12^C^16^O, ^17^O^12^C^16^O, with natural abundances of 0.984204, 0.0110574, 0.00394707, and 0.000733989, respectively. Exhaled CO_2_ is usually a product of glucose catabolism in the human body, and previous research revealed that carbon-13 (^13^C) in breath CO_2_ has a potential link with the gastric pathogen *Helicobacter pylori* (*H. pylori*) in response to glucose ingestion [6,7]. *H. pylori* is widely acknowledged to be associated with a variety of clinical outcomes, such as duodenal ulcer, gastric ulcer, distal gastric adenocarcinoma, and gastric mucosa associated lymphoid tissue lymphoma [8,9,10]. In addition, a growing body of evidence suggests that the effects of *H. pylori* may also be relevant to several other extragastric diseases including idiopathic thrombocytopenic purpura, cardiovascular disease, anemia, diabetes, and insulin resistance [11,12]. In recent years, a few studies have reported that the oxygen-18 (^18^O) isotopes in exhaled CO_2_ are also a biomarker related to *H. pylori* because of the rapid exchange of the ^16^O in ^12^C^16^O_2_ and ^18^O in H_2_^18^O in response to periplasmic *α*-carbonic anhydrase activity [13,14,15].

For gas identification and quantification, there are significant advantages to laser absorption spectroscopy, such as high sensitivity, high selectivity, fast response, and real-time online analysis [16,17,18,19,20]. Many laser absorption spectroscopy based sensors have been developed for monitoring the human breath CO_2_ isotopes in recent years, especially for ^13^C. Generally, the *δ* value has been used to describe the relative deviation of the sample isotope ratio from the standard reference value (e.g., the standard abundance in Vienna Peedee Belemnite (VPDB)). Crosson et al. constructed a cavity ring-down spectrometer using a three-mirror high-finesse ring-down cavity, and demonstrated the ability to obtain *δ*^13^C in breath samples [21]. Kasyutich et al. developed an off-axis cavity-enhanced absorption spectrometer, and estimated *δ*^13^C standard deviations of 1.8‰ and 3.7‰ based on peak height and integrated area estimations, respectively [22]. A multidimensional linear regression technique to calculate the *δ*^13^C isotope was reported by Andreev et al., and precision of 0.07‰ was obtained at an averaging time of 3 min thanks to a Herriott multipass cell [23]. Based on an off-axis integrated cavity output spectroscopy system, Pradhan’s group studied the mechanisms linking exhaled *δ*^13^C and *δ*^18^O of CO_2_ to *H. pylori* [12,13,14,15]. These measurements at near infrared require the assistance of a high-finesse optical cavity or a multipath cell, which is usually accompanied by a larger volume of gas demand, so they are not really friendly for real-time online breath analysis, where the sample gas is usually limited. A hollow waveguide with a small volume was used by Zhou et al. to achieve simultaneous measurement of *δ*^13^C and *δ*^18^O, and the minimum precisions were 0.26‰ and 0.57‰ for *δ*^13^C and *δ*^18^O, respectively, achieved by calibration-free wavelength modulation spectroscopy [24,25]. However, there is currently no measurement of *δ*^17^O in exhaled CO_2_, although studies have shown that ^17^O isotopes in respiration can be used as a biomarker of brain oxygen metabolism [26,27].

In this paper, a breath diagnosis system has been developed for simultaneous analysis of *δ*^13^C, *δ*^18^O, and *δ*^17^O in human breath CO_2_ based on mid-infrared laser direct absorption spectroscopy employing an interband cascade laser (ICL) at 4.33 μm. Considering the practical application of the system, a single path cell with a small volume was adopted, and the design of multi-channel gas sampling can meet the needs of real-time measurement and batch processing. The accuracy of the system was improved by the high-precision control of temperature and pressure.

## 2. Isotopes Experimental Theory

For laser direct absorption spectroscopy, the absorption at frequency *ν* is given by the Beer–Lambert law

−ln(*I*_T_(*ν*)/*I*_0_(*ν*)) = *Nσ*(*ν*)*L*, (1)

where *I*_0_(*ν*) and *I*_T_(*ν*) are incident and transmitted laser intensity, respectively, *N* (molecules/cm^3^) is the number density of absorbing molecules, *L* (cm) is the absorption path length, and *σ*(*ν*) (cm^2^/molecule) is the absorption cross section related to frequency. The integrated *σ*(*ν*) is equal to the temperature-dependent transition intensity *S*(*T*) (cm^−2^/atm) and another isotopologue abundance *n* needs to be considered for isotopes measurement. Thus, the integral absorption area *A* can be expressed as:

*A* = ∫ *Nσ*(*ν*)*L*
*dν* = *NLS*(*T*)/*n*. (2)

For isotope ratio calculation, it can be directly expressed by the ratio of molecular number densities:

(3) $$R=\frac{N_{r}}{N_{a}}=\frac{A_{r}n_{r}{/S}_{r}(T)L}{A_{a}n_{a}{/S}_{a}(T)L}=\frac{A_{r}}{A_{a}}\cdot \frac{S_{a}{(T)/n}_{a}}{S_{r}{(T)/n}_{r}},$$

where in r and a represent rare isotopic species (^13^C^16^O_2_, ^18^O^12^C^16^O and ^17^O^12^C^16^O) and abundant isotopic component (^12^C^16^O_2_), respectively. In this work, the VPDB standard is adopted to express the isotope ratios as *δ* values, and the $R_{{\text{VPDB-CO}}_{2}}^{13}$, $R_{{\text{VPDB-CO}}_{2}}^{18}$, and $R_{{\text{VPDB-CO}}_{2}}^{17}$ are 0.011180, 0.0003931, and 0.00208835, respectively. Thus, the sample gas stable isotope *δ* value is given by:

(4) $$\delta =\left(\frac{R}{R_{\mathrm{VPDB}}}-1\right)\times 1000‰.$$

As can be seen in Equation (3), except for the experimentally measured integral absorption area, the isotope ratio is only related to the transition intensity and isotopologue abundance *n*. It should be noted that *n* is a constant, and the temperature-dependent line strength is given by [28]:

(5) $$S(T)=S(T_{0})\frac{Q(T_{0})}{Q(T)}\exp[-\frac{hcE^{\prime \prime }}{k}(\frac{1}{T}-\frac{1}{T_{0}})]\times [-\exp(1-\frac{hcv_{0}}{kT})]{\left[1-\exp(-\frac{hcv_{0}}{kT_{0}})\right]}^{-1}$$

where *S*(*T*_0_) is the line strength at reference temperature *T*_0_ (usually *T*_0_ = 296 K), *Q*(*T*_0_) and *Q*(*T*) are the partition functions of the absorbing molecule, *h* (J s) is the Planck’s constant, *c* (cm/s) is the speed of light, *k* (J/K) is the Boltzmann’s constant, *E*″ (cm^−1^) is the lower state energy and *ν*_0_ (cm^−1^) is the transition center frequency. The temperature stability will also affect the *δ* value measurements, and this temperature dependence Δ*δ*/Δ*T* is proportional to the difference of ground state energies of the transitions [29]:

(6) $$\frac{\Delta \delta }{\Delta T}\approx \frac{\Delta E^{\prime \prime }}{kT^{2}}\times 1000‰.$$

## 3. Sensor Structure and Optimization

### 3.1. Transitions Selection

**Figure 1:**
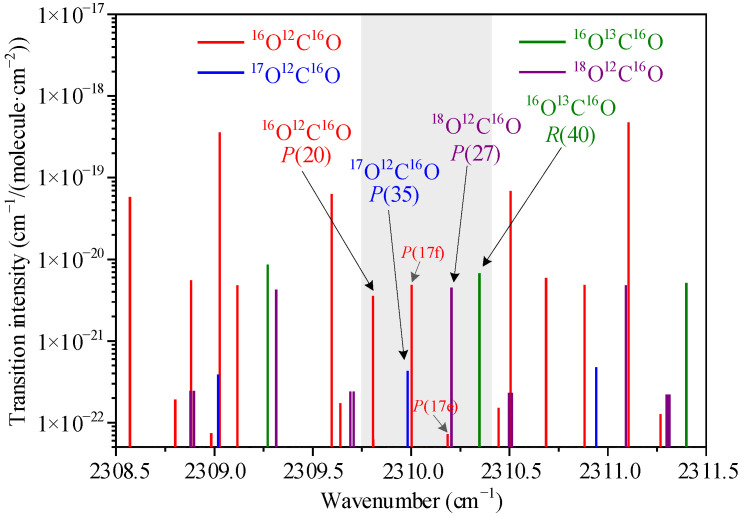
Absorption lines of CO_2_ isotopes at 4.33 μm based on HITRAN 2020.

For the linearly symmetrical CO_2_ molecule, there is a strong antisymmetric stretching vibration (*ν*_3_) at 4.3 μm, and the transition intensity here is several orders of magnitude greater than that in near infrared. In this paper, the transitions of the *P*(20) line of the ^16^O^12^C^16^O (1, 0^0^, 11) ← (1, 0^0^, 1) band at 2309.81 cm^−1^, the *R*(40) line of the ^16^O^13^C^16^O (0, 0^0^, 11) ← (0, 0^0^, 1) band at 2310.35 cm^−1^, the *P*(27) line of the ^18^O^12^C^16^O (0, 0^0^, 11) ← (0, 0^0^, 1) band at 2310.21 cm^−1^, and the *P*(35) line of the ^17^O^12^C^16^O (0, 0^0^, 11) ← (0, 0^0^, 1) band at 2309.98 cm^−1^ were selected based on the HITRAN database [30]. As shown in the shadow of Figure 1, there are similar transition intensities (weighted by isotopologue abundance) with tolerable gaps, and the span of the selected transitions is less than 1 cm^−1^, which can be detected simultaneously by a single laser current sweep. In addition, there is no absorption disturbance from other exhaled substances such as H_2_O, O_2_, and other trace gases. It should be noted that low pressure is required to avoid the overlap of spectral lines caused by pressure broadening, especially for the ^17^O^12^C^16^O transition and the *P*(17f) line of the ^16^O^12^C^16^O (0, 2^2^, 11) ← (0, 2^2^, 1) band at 2310.00 cm^−1^. Although there is a *P*(17e) line of the ^16^O^12^C^16^O (0, 0^0^, 21) ← (0, 0^0^, 11) band at 2310.19 cm^−1^, it has less effect on the next ^18^O^12^C^16^O *P*(27) line due to the low transition strength.

### 3.2. Experimental Setup

**Figure 2:**
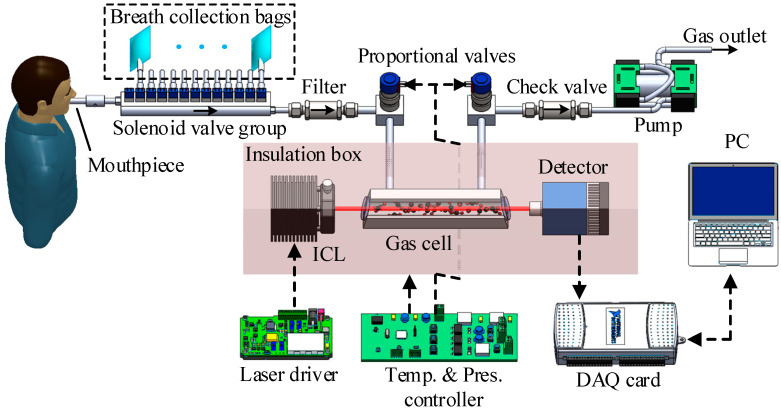
Schematic diagram of the developed sensor for simultaneous measurement of ^12^C^16^O_2_, ^13^C^16^O_2_, ^18^O^12^C^16^O and ^17^O^12^C^16^O in human breath.

Figure 2 shows the schematic diagram of the developed sensor for simultaneous analysis of human breath CO_2_ isotopes. A room temperature ICL (manufactured by Nanoplus, Gerbrunn, Germany) emitted at 4.33 μm was used as the laser source, and its working temperature and scanning current were controlled by a homemade mid-infrared laser controller. The radiated laser light was firstly collimated through the gas cell and then focused on the thermoelectrically cooled mercury cadmium telluride photodetector (VIGO system). Finally, the spectrum signal was acquired by the data acquisition (DAQ) card and processed by PC.

All the optical components and gas cell were thermally insulated. With the feedback from a 10 kΩ thermistor mounted onto the gas cell, a pair of thermoelectric coolers controlled by a homemade temperature controller based on digital PID algorithm were adopted to regulate the temperature in the insulation case.

Since breath analysis has the feature of less sample gas, especially for real-time measurement, a single pass cell with a small volume was adopted in the system. The length of the gas cell was 150 mm, with an inner diameter of 6 mm, which results in a volume of less than 5 mL. The gas inlet was controlled by a solenoid valve group so that the sensor can work in real-time measurement and batch mode. Real-time measurement is to directly sample the exhaled breath gas through the replaceable mouthpiece. The batch mode can automatically process multiple prepared breath collection bags, and 12 channels were included in this system, which is more convenient when it is needed to compare the isotope abundance difference before and after taking medicine or when there are a lot of samples. The breath sample was first filtered out of water and particles and then extracted to the gas cell by a pump. A check valve was placed behind the cell to prevent backflow. Two solenoid operated proportional valves were equipped to control the inlet and outlet flow rates to ensure a stable low pressure in the gas cell, combined with a pressure transducer and digital PID algorithm based homemade pressure controller.

### 3.3. Spectrometer Optimization

**Figure 3:**
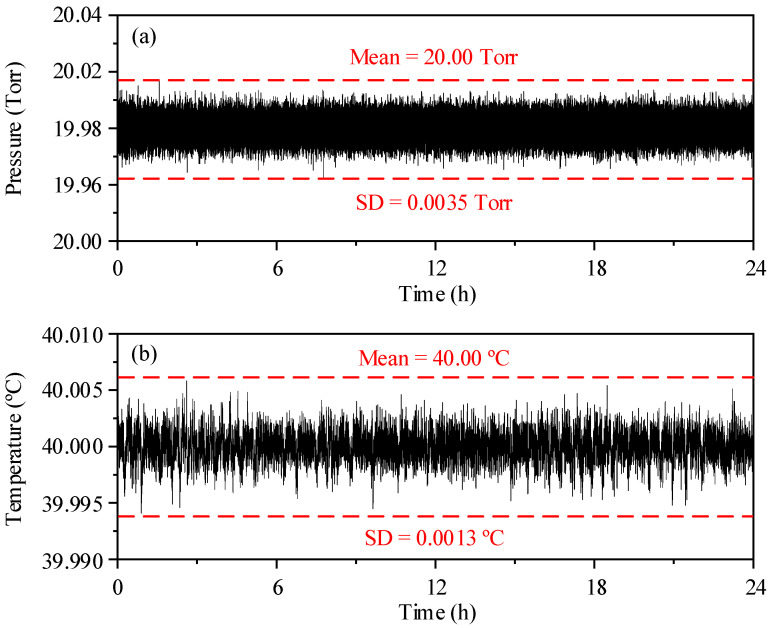
(**a**) Pressure in gas cell and (**b**) temperature in insulation case recorded continuously a 24 h period.

Although the isotope abundance measurement based upon the direct absorption peak areas is theoretically immune to the pressure variation in the optical cell, a stable pressure can reduce the unnecessary errors in the optical baseline removal and signal fitting. The pressure in the gas cell was set at 20 Torr to weaken the overlap of the spectra while further reducing the sample gas consumption. The continuous 24 h data of the pressure in the gas cell were recorded and displayed in Figure 3a, the mean value is consistent with the set value, and the standard deviation of the data is 0.0035 Torr.

According to Equation (6), theoretical temperature coefficients Δ*δ*^13^C/Δ*T* = 15.0‰ K^−1^, Δ*δ*^18^O/Δ*T* = 20.9‰ K^−1^, and Δ*δ*^17^O/Δ*T* = 17.7‰ K^−1^ are estimated at 296 K for the transitions chosen in this work. To minimize the temperature caused drifts, the temperature of the gas cell and optical components were precisely controlled. The target temperature was set at 40 °C, which is higher than room temperature for a better result. Figure 3b displays the continuously recorded 24-h temperature from the thermistor, the mean value is consistent with the target, and the standard deviation is 0.0013 °C, which result in a possible *δ*-value drifts of only 0.017‰, 0.024‰, and 0.021‰ for *δ*^13^C, *δ*^18^O, and *δ*^17^O, respectively. It provides an important guarantee for the accuracy and precision of the sensor system.

**Figure 4:**
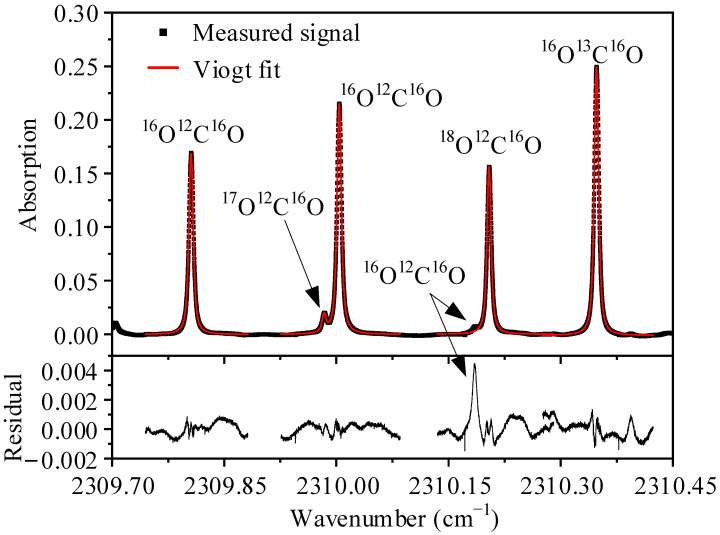
Measured absorption signals fitted to Voigt profile and fitting residuals.

**Figure 5:**
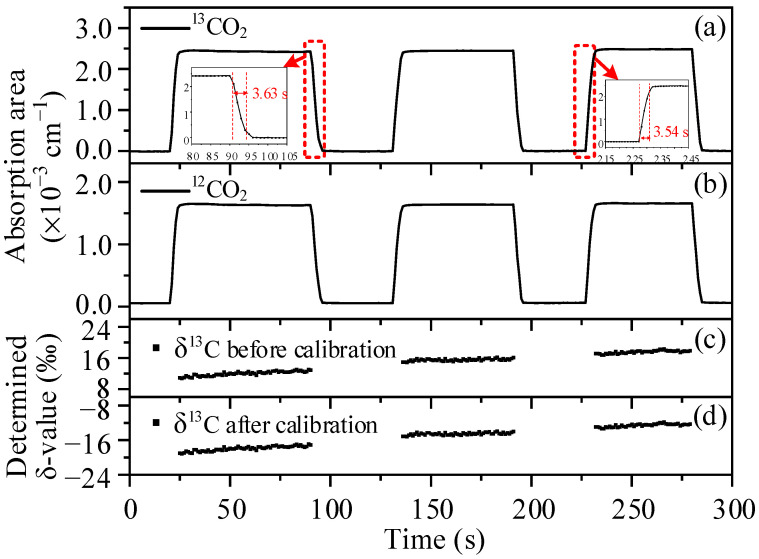
(**a**) Measured ^13^CO_2_ absorption areas and (**b**) measured ^12^CO_2_ absorption areas when the developed system alternately measures ambient air and cylinder gases with *δ*^13^C values of −18.5‰, −15.5‰, and −12.5‰. (**c**) Calculated *δ*^13^C from absorption areas and uncalibrated temperature value. (**d**) Calculated *δ*^13^C based on calibrated temperature value.

**Figure 6:**
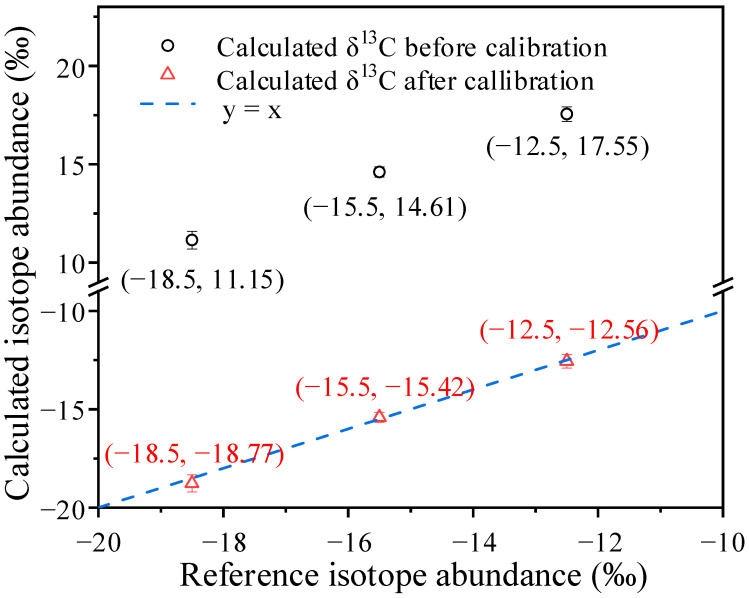
The relationship between the calculated *δ*^13^C values and the reference values.

To determine the accurate isotope abundance, the absolute temperature value is also an important factor, which can be found from Equations (3) and (5). Although there is a great accuracy of temperature controlling, its absolute value still needs to be calibrated. In this work, three cylinder gases with known *δ*^13^C values (−18.5‰, −15.5‰, and −12.5‰) were used to calibrate the absolute temperature in the incubator. Figure 4 shows the measured absorption signal of the gas with *δ*^13^C of −15.5‰, and the optical baseline was removed by the least square fitting of the light intensity without absorption. To obtain the integral absorption areas, the signal is divided into four segments to fit the Viogt profile respectively. It should be noted that the double peak fitting has to be used for the ^17^O^12^C^16^O peak and the next *P*(17f) ^16^O^12^C^16^O peak, conversely the single peak fitting is used for ^18^O^12^C^16^O due to the negligible effect from the *P*(17e) ^16^O^12^C^16^O peak. Obviously the measured absorptions match well with the Viogt fitting data, and the residuals are less than ±1‰ except for the unfitted ^16^O^12^C^16^O peak at 2310.19 cm^−1^. As shown in Figure 5a,b, the initial measurement gas was ambient air, and then alternate measurement takes place between the three cylinder gases and ambient air. The results indicate a 90–10% fall time of about 3.63 s and a 10–90% rise time of about 3.54 s, thanks to the small volume of the single pass absorption cell. As expected, the calculated *δ*^13^C values before temperature calibration are far from the nominal values as exhibited in Figure 5c and Figure 6; however, the difference can be reduced by changing the temperature value, and an optimal temperature of 310.93 K was adopted. The calculated *δ*^13^C values with corrected temperature are displayed in Figure 5d and Figure 6, they are exactly close to the nominal value, and the error is less than 0.3‰. Therefore, in subsequent calculations, the absolute temperature value is considered to be 310.93 K, and the absolute *δ*^13^C, *δ*^18^O, and *δ*^17^O can be calculated from Equation (3).

## 4. Sensor Performance

### 4.1. Concentration Dependence

**Figure 7:**
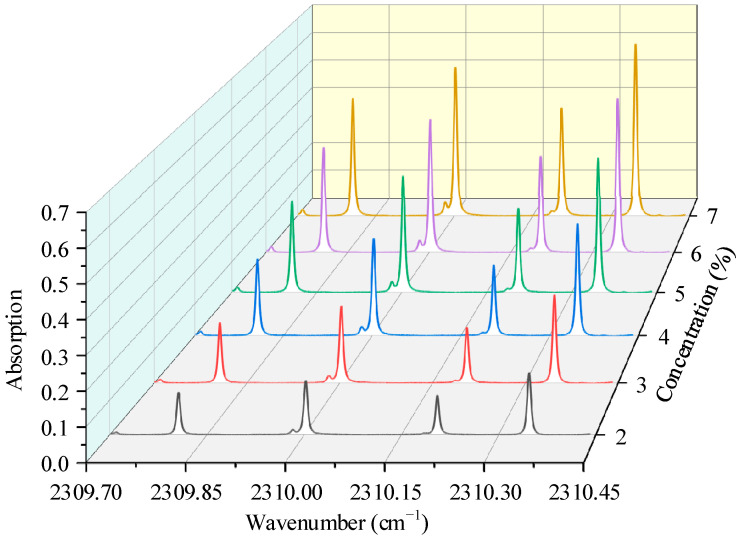
Measured absorption signals of CO_2_ stable isotopes with a total concentration of 2–7%.

**Figure 8:**
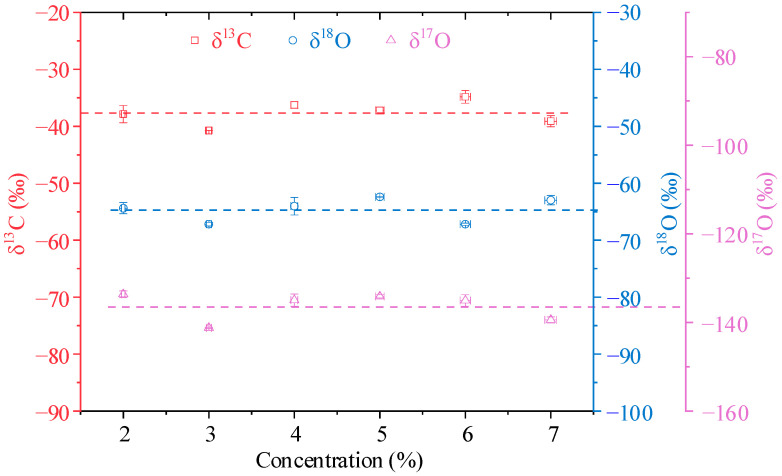
Calculated isotopic abundances of the diluted CO_2_ gases with a total concentration of 2–7%.

Figure 7 displays the CO_2_ absorption spectrum signals measured by the developed system. The CO_2_ sample gases with a concentration range of 2–7%, which covers the exhaled breath CO_2_ concentration, were prepared by diluting pure CO_2_ with N_2_. As shown in Figure 7, the weakest absorption peak of ^17^O^12^C^16^O can still be clearly distinguished with a signal-to-noise ratio of 8 even when the sample CO_2_ concentration is 2%. The calculated isotopic abundances of the different diluted gases are shown in Figure 8, they are evenly distributed around certain values, and which is following the theory that the isotope abundance does not depend on gas concentration. Part of the calculation result deviation may come from the baseline removal and fitting error caused by a wide range of concentration changes.

### 4.2. Stability and Detection Limit

**Figure 9:**
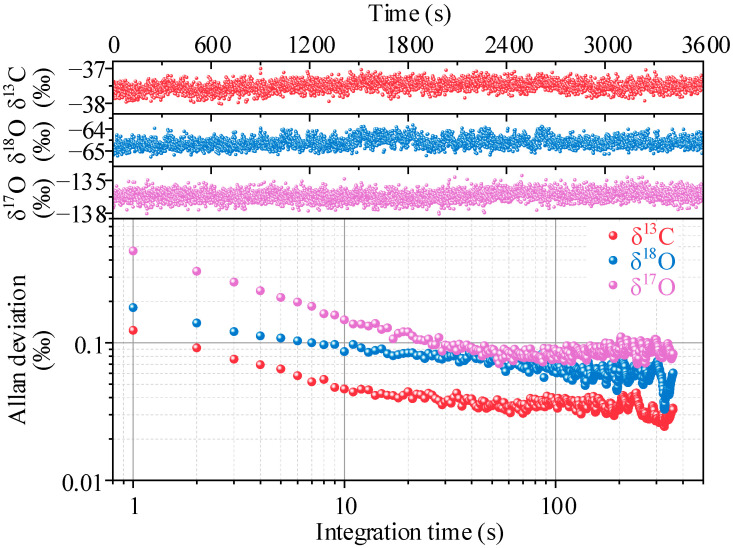
Raw measurement results of *δ*^13^C, *δ*^18^O, and *δ*^17^O (**upper panels**) and Allan deviation plot as a function of integration time (**lower panel**).

The stability and minimum detection limits of the sensor system were evaluated by Allan variance. The 5% CO_2_ gas with stable isotopes (*δ*^13^C = −37.51‰, *δ*^18^O = −64.61‰, and *δ*^17^O = −136.4‰) were measured at 1 Hz for 1 h, the recorded raw data of *δ*^13^C, *δ*^18^O, and *δ*^17^O are plotted in the upper panels of Figure 9, and the lower panel shows the Allan variance as a function of the measurement time. As shown in the Allan deviation, the precisions of the system at 1 s are 0.12‰, 0.18‰, and 0.47‰ for *δ*^13^C, *δ*^18^O, and *δ*^17^O, respectively. When the integration time is short, the theoretically expected behavior of the system mainly comes from white noise. At the integration time of 10 s, the precisions of the system can be optimized as 0.046‰, 0.086‰, and 0.15‰ for *δ*^13^C, *δ*^18^O, and *δ*^17^O, respectively.

### 4.3. Real-Time Measurment of Human Breath

**Figure 10:**
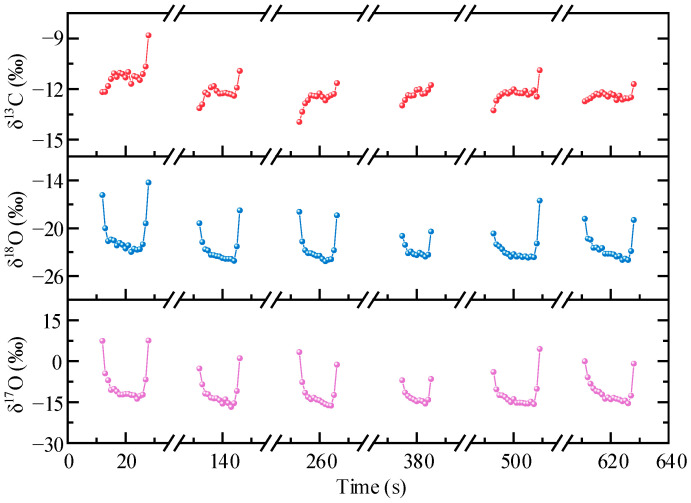
Real-time measurement of the exhaled *δ*^13^C, *δ*^18^O, and *δ*^17^O.

Real-time measurement was performed to detect the CO_2_ stable isotope abundances in the breath. Figure 10 shows the analysis results of six repeated exhalations. The interval between each breath analysis was about 2 min so that there was a regular breathing rate and there was no residual gas. In the analysis results, there are large fluctuations in the start and end positions of each cycle. This is mainly due to the incomplete gas replacement in the gas cell at the start and end of the measurement process. The isotope abundances averaged from the reliable values in the middle of the six breathing cycles are (−12.12 ± 0.22)‰, (−23.35 ± 0.26)‰, and (−14.19 ± 0.56)‰ for *δ*^13^C, *δ*^18^O, and *δ*^17^O, respectively. The deviations are slightly larger than Allan deviations, maybe because of the instability of the airflow in real-time breath measurement.

## 5. Discussion

In this paper, we report a breath gas sensor for simultaneous measurement of *δ*^13^C, *δ*^18^O, and *δ*^17^O in CO_2_. The carefully selected transitions were covered by a single ICL emitted at 4.33 μm. A gas cell with a volume less than 5 mL was equipped, which is friendly to exhalation diagnosis with less sample gas. In addition to real-time breath sampling, a batch mode was also included that can automatically process multiple prepared gas sampling bags. To reduce the unnecessary errors, the pressure in the gas cell was precisely controlled with a standard deviation of 0.0013 Torr. Moreover, the temperature in the insulation case was precisely controlled with a precision of 0.0013 °C, resulting in small temperature dependence drifts of 0.017‰, 0.024‰, and 0.021‰ for *δ*^13^C, *δ*^18^O, and *δ*^17^O, respectively. An error of not more than 0.3‰ was found between the system responded *δ*-value and the reference. Precisions of 0.12‰, 0.18‰, and 0.47‰ were estimated at 1 s integration time for *δ*^13^C, *δ*^18^O, and *δ*^17^O, respectively, from Allan variance, and they can be optimized to 0.046‰, 0.086‰, and 0.15‰ at 10 s integration time, respectively. Finally, to demonstrate the potential for disease diagnosis, similar precisions were obtained in real-time breath measurement. Compared with the gas sensor in recent work [24] mentioned in the introduction section, our system reaches a more than five times better precision on *δ*^13^C and *δ*^18^O measurements. The obtained results highlight the viability of the laser spectroscopic system in general and its potential for practical application in breath analysis.

The ^17^O isotope is considered the least abundant stable isotope of oxygen; however, since it is the only oxygen nuclei with a magnetic moment, ^17^O-labeled H_2_O (H_2_^17^O) has been measured by magnetic resonance imaging (MRI) for studying the oxidative metabolism in brains which is connected to many diseases such as schizophrenia, Alzheimer’s disease, Parkinson’s disease, or the process of aging [26,27]. The H_2_^17^O molecules used for MRI are products of the respiration of ^17^O-labeled oxygen molecules in mitochondria. Studies have shown that the oxygen isotopes in CO_2_ and H_2_O will exchange rapidly through a bicarbonate ion when catalyzed by carbonic anhydrase in whole blood [31,32,33], so there will be theoretical isotopic enrichments of ^17^O in breath CO_2_ when a dose of ^17^O-labeled oxygen is ingested, and the ^17^O isotope in breath CO_2_ may be related to cerebral oxygen metabolism, which is similar to the mechanisms linking metabolism of *H. pylori* to ^18^O-isotopes of human breath CO_2_. Moreover, ^18^O and ^17^O isotopes in breath CO_2_ may be potential biomarkers for the early infection detection or preclinical stage of diseases related to both *H. pylori* and oxygen metabolism. However, to determine whether ^17^O of exhaled CO_2_ is related to *H. pylori* and oxygen metabolism, sufficient and credible samples are required, which is limited by patients and isotope labeled urea in this paper.

## 6. Conclusions

This work has shown a potentially valuable laser spectroscopic system that can simultaneously obtain valid data of *δ*^13^C, *δ*^18^O, and *δ*^17^O in exhaled CO_2_. Simultaneously sensitive measurement of the *δ*^13^C, *δ*^18^O, and *δ*^17^O in human exhaled CO_2_ can provide a more comprehensive reference for metabolic status monitoring or disease diagnosis. It would be useful for non-invasive detection of different diseases in the human body. However, future works are still needed for the implementation, especially the experimental determination, of the specific link between O isotopes in CO_2_ and *H. pylori* or oxygen metabolism. Besides, it would be interesting to measure the breath samples at different stages of diseases.
